# Two confirmed cases of severe fever with thrombocytopenia syndrome with pneumonia: implication for a family cluster in East China

**DOI:** 10.1186/s12879-017-2645-9

**Published:** 2017-08-03

**Authors:** Yiyi Zhu, Huanyu Wu, Jie Gao, Xin Zhou, Renyi Zhu, Chunzhe Zhang, Hongling Bai, Abu S. Abdullah, Hao Pan

**Affiliations:** 1 Shanghai Municipal Center for Disease Control and Prevention, Shanghai, 200336 China; 2 Shanghai Jingan Center for Disease Control and Prevention, Shanghai, 200072 China; 3 Global Health Program, Duke Kunshan University, Kunshan, Jiangsu Province 215347 China; 4 Duke Global Health Institute, Duke University, Durham, NC 27710 USA; 5 Department of General Internal Medicine, School of Medicine, Boston University Medical Center, Boston, MA 02118 USA

**Keywords:** Severe fever with thrombocytopenia syndrome, New bunyavirus, Human-to-human transmission, Family cluster

## Abstract

**Background:**

Severe fever with thrombocytopenia syndrome (SFTS) was first reported in China in 2011. Human-to-human transmission of the virus occurred occasionally in family clusters. However, pneumonia as an onset syndrome was not common in most SFTS cases. Our aim is to report a family cluster of SFTS with clinical manifestation of pneumonia in Shanghai.

**Methods:**

Epidemiologic investigations were conducted when a family cluster of severe fever with thrombocytopenia syndrome virus (SFTSV) infection was identified in Shanghai in June 2016. Samples were collected from two secondary cases and two close contacts with fever. SFTSV was detected by Real-Time reverse transcription polymerase chain reaction (RT-PCR).

**Results:**

There were two confirmed STFS cases and one potential index case. The potential index case became ill on 21 May and died on 31 May. Case A had onset from 4 to 23 June and case B from 8 June to 25 June. All the three cases experienced pneumonia at the early stage of SFTSV infection. Three (3) out of thirty two (32) close contacts had symptoms of fever or cough but were detected STFSV negative by real-time RT-PCR. According to epidemiologic investigations, the potential index case had outdoor activities on a nearby hill. A tick bite could have been the reason for the SFTSV infection in the potential index case as ticks were found both in grassland or shrubs on the hill and also found on mice caught in her house. Both cases A and B had provided bedside care for the potential index case without any protection and had contacted with blood and other body fluids.

**Conclusion:**

It was a family cluster of SFTSV infection imported from Jiangsu province located in the east of China. We suggested to become alert to atypical SFTSV infected cases.

## Background

An emerging infectious disease, severe fever with thrombocytopenia syndrome (SFTS) was first reported in China in 2011 [1]. A pathogen causing SFTS was simultaneously identified as a novel Bunyavirus, designated SFTSV. Till now, increasing number of new cases have also been reported in Japan, Korea. In Japan, 163 patients with SFTS were confirmed from the autumn of 2012 and October 2015 [2]. Most cases occurred in the western part of Japan. The numbers of SFTS cases also increased annually from 36 (2013), 55 (2014) and 79 (2015) in the Republic of Korea [3]. 5,352 SFTS cases were reported in 23 provinces in China from 2011 to 2014. 99.3% cases were reported from Henan, Shandong, Hubei, Anhui, Liaonin, Zhejiang, and Jiangsu provinces [4]., SFTS cases in China significantly increased from 2013 to October 2016, 7419 cases with 355 deaths. Since 2014, over 1000 SFTS cases were reported annually in Anhui, Henan, Hubei, and Shandong Provinces [5]. Zhejiang and Jiangsu provinces are both adjacent to Shanghai. A reported case of SFTS in Shanghai was imported from Anhui province, which was historically the only reported SFTS case in Shanghai [6].

Human-to-human transmission of the virus occurred occasionally in family clusters. Several family cluster cases were reported previously in China and Korea [7–12]. Contact with the index patient’s blood was significantly associated with development of SFTS. In systematic studies SFTS case fatality rate (CFR) was 12–16% and major clinical features were fever, thrombocytopenia, leucopenia, gastrointestinal symptoms and central nervous system manifestations [13, 14].

We reported a family cluster from Suzhou, Jiangsu Province of China, which was confirmed in Shanghai. Three members of a family presented with high fever and pneumonia were admitted and two of them were detected to be SFTSV positive. Pneumonia was an atypical syndrome for SFTS cases. Epidemiological investigation, clinical syndromes and symptoms, and laboratory testing were described and more evidence of transmission mechanism were provided to further understand SFTS.

## Methods

### Epidemiological investigation

On June 13, three cases were reported to Shanghai Municipal Center for Disease Control and Prevention (SCDC) and were interviewed immediately by Shanghai local CDC staff. Basic demographic information, clinical manifestation, epidemiological history and timeline of the cluster were collected and analyzed. Exposure to outdoor activities referring to work, live or travel in the regions of hills, forests and mountains in main epidemic season 2 weeks before onset was inquired. Epidemiological investigations were also carried out to obtain information for close contacts, defined as anyone who had contacted with blood, fluid, bloody secretion or excretion of the SFTS patients without any protection. Fourteen-day medical observations among close contacts were initiated on June 14 [15]. Environmental investigation was carried out immediately to identify ticks around the residential areas of cases. The tick specimens were collected by flagging white cloth on grasslands and also picking from animals’ body surface.

### Sample collection

The index patient died before the family cluster was identified and therefore there was no sample available for further testing. Two secondary patients and close contacts with fever symptoms during the first week of contact were sampled.

### Laboratory analysis

Nucleic acid of SFTS was detected from serum specimens of case A (daughter of the potential index case), B (the son of the potential index case), C (granddaughter of the potential index case), as well as close contacts with fever and other symptoms (Tables 1 and 2). Real-Time Reverse Transcriptase Polymerase Chain Reaction (RT-PCR) [16] was adopted in testing SFTSV in serum. RNA was extracted from serum by Total Nucleic Acid Isolation kit (Roche Diagnostics) according to the manufacturer’s instruction. SFTS viral segments were amplified by primers and probes (provided by China CDC). Real-time PCR was performed as follows: 50 °C for 30 min, 95 °C for 10 min. Then 40 cycles of amplification was undertaken: 95 °C for 15 s and 60 °C for 45 s. The cutoff cycle threshold (C_t_) value for a positive sample was at 35 cycles. A C_t_ value less than 35 was judged as positive.

**Table 1:** Potential index case and secondary cases in this family cluster of SFTSV

Case	Gender	Age	Relation with index case	Onset date	Onset symptoms	Blood routine testing (admitted)	Liver toxicity Testing (admitted)	Treatments	Real Time RT-PCR	Outcome
Potential index case	Female	73	-	2016/5/21	Fever, malaise	WBC1.83 × 10^9^/L PLT38 × 10^9^/L	ALT220.0 U/L AST805.4 U/L	Cefmetaz	Not available	Deceased
A	Female	47	Daughter	2016/6/4	Fever, cough, malaise	WBC2.29 × 10^9^/L PLT97 × 10^9^/L	ALT 67.0 U/L AST59.0 U/L	Ofloxacin, Methylprednisolone Sodium Succinate, Pantoprazole, Glutamine, Xiyanping	Positive	Cured
B	Male	53	Son	2016/6/8	Fever, malaise	WBC1.78 × 10^9^/L PLT56 × 10^9^/L	ALT 56.0 U/L AST122.0 U/L	Piperacillin, Methylprednisolone Sodium Succinate, Pantoprazole, Glutamine, Xiyanping	Positive	Cured

**Table 2:** Close contacts with fever and other symptoms in this family cluster of SFTSV

Case	Gender	Age	Relation with the potential index case	Onset date	Onset symptoms	Real time RTPCR detection	Outcome
C	Female	30	Granddaughter	2016/6/1	Fever, cough	Negative	Cured
D	Male	66	Brother-in-law	2016/6/8	Fever	Negative	Cured
E	Male	65	Funeral service	2016/6/9	Fever, malaise	Negative	Cured

## Results

### Case A

**Fig. 1:**
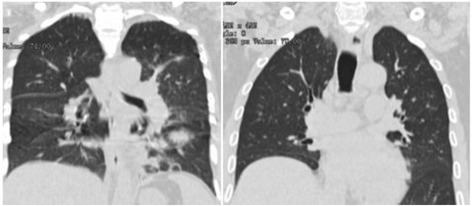
Computed Tomographic Scans of the Chest of case A and case B

Case A was a 47-year-old female. On June 4, 2016, she began to feel sick. Next day, she found herself with high fever of 39.9 °C, coughing, sore throat and malaise. She visited local hospitals A and B in Jiangsu Province. Then she was admitted by hospital B and treated with Ticarcillin/Clavulanate Potassium and levofloxacin. No sign of improvement was observed. Laboratory analysis of blood revealed leukopenia (white blood cells count 2.29 × 10^9^/L) and thrombocytopenia (platelets count 97 × 10^9^/L). Occult blood (25 + cell/u) and albumin (80 mg/L) were found in urine routine testing. In biochemistry testing, lower total protein (61.5 g/l), pre-albumin (126 mg/l), and elevated blood sugar was detected. Antibiotics, Oseltamivir and Insulin were administrated to control infection and lower the blood sugar. On June 11, blood testing still showed leukopenia (white blood cells count 2.42 × 10^9^/L) and thrombocytopenia (platelets count 68 × 10^9^/L). Mycobacterium tuberculosis (TB), EB virus, Cox A16 virus, EV71 virus, *Chlamydia pneumoniae*, syncytial virus, adenovirus, influenza virus and para-influenza virus were all detected as negative. The case was then transferred to hospital B in Shanghai on the same day. Presenting with the symptoms of coughing and malaise and with chest computerized tomography (CT) of “Left lung patchy shadow, bilateral small amount of pleural effusion, increased width of the longitudinal diaphragm” (Fig. 1), case A was admitted in hospital C. Thrombocytopenia (platelets count 81 × 10^9^/L) continued and white cell counts were normal. Rapid testing for influenza A was negative. Alanine aminotransferase (ALT) and aspartate aminotransferase (AST) were elevated as 67.0 U/L and 59.0 U/L respectively. Ofloxacin Capsules, Methylprednisolone Sodium Succinate, Pantoprazole, Glutamine and Xiyanping (antiviral herbal medicine) were prescribed. Case A’s temperature got normal. Cough and muscle soreness were relieved after treatment.

### Case B

Case B was case A’s elder brother. He was a 53-year-old and became sick since June 8, 2016. Symptoms including fever (38.5 °C), malaise and low lumber soreness appeared early during his illness. He visited hospital B in Jiangsu province on June 9, 2016. He was treated with Reduning (Antiviral herbal medicine), Oseltamivir, Levofloxacin, Ticarcillin/ Clavulanate Potassium and Insulin aspart. Blood routine testing upon admission showed leukopenia (white blood cells count 2.29 × 10^9^/L), erythropenia (red cell count 4.10 × 10^12^/L), thrombocytopenia (platelets count 56 × 10^9^/L), occult blood in fecal sample, elevated ALT (56.0 U/L) and AST (122.0 U/L), lowered total protein (59.4 g/L), lowered albumin (38.2 g/L), pre-albumin (163 mg/L), elevated lactate dehydrogenase (LDH) 240 U/L, and elevated blood sugar (12.84 mmol/L). X-ray detection showed increased bronchovascular shadows. He was also screened for other pathogens such as Mycobacterium TB, EB virus, Cox A16 virus, EV71 virus, *Chlamydia pneumoniae*, syncytial virus, adenovirus, influenza virus and para-influenza virus, but all tests were negative. Together with case A, he was transferred to hospital C in Shanghai and was admitted for viral pneumonia and type II diabetes. CT showed two nodular shadows in the left lung and pleural effusion in the right lung (Fig. 1). A Swab was collected and a test for Influenza A showed negative results. Blood routine testing still showed leukopenia (white blood cells count 3 × 10^9^/L) and thrombocytopenia (platelets count 53 × 10^9^/L). Blood gas analysis revealed lowered partial pressure of carbon dioxide (PCO_2_) (4.2kpa) and total carbon dioxide (TCO_2_) (22.3 mmol/L). Coagulopathy (activated partial thromboplastin time (APTT) 46.8 s) and elevated creatine phosphate kinase (260 IU/L) was also observed. Whole blood testing was done again on June 13. Leukopenia (white blood cells count 2.3 × 10^9^/L) and thrombocytopenia (platelets count 28 × 10^9^/L) had worsened.

### Potential index case

The potential index case was a 72-year-old woman. She was the mother to cases A and B. Illness of potential index case began on May 21, 2016. She was sent to a local clinic by case A on the same day. Blood testing showed leukopenia (white blood cells count 3.83 × 10^9^/L) and elevated blood sugar. She was treated for viral infection. On May 23, she had worse symptoms of fever (39 °C), bleeding gums, stomachache, diarrhea and malaise. Again, case A took her to a local community health center for treatment. Blood testing showed leucopenia (white blood cells count 2.38 × 10^9^/L) and thrombocytopenia (platelets count 88 × 10^9^/L). Dermal ecchymosis appeared on the index case’s chest and upper lumber. On May 25, she felt nausea and vomited. She visited hospital D in Jiangsu province. Blood testing showed leukopenia (white blood cells count 1.83 × 10^9^/L), thrombocytopenia (platelets count 32 × 10^9^/L), elevated liver-associated enzyme levels (AST 805.4 U/L; ALT 220.0 U/L, coagulopathy (Prothrombin Time 13.7 s and APTT 64.1 s). Urine testing showed abnormal sugar (++), protein (++), occult blood (+++). Heteropathy was applied. However, she got worse and began convulsing. She died of multiple-organ failure on May 28. According to the guideline for prevention and treatment of SFTS [17], she was confirmed as a probable case of SFTS.

### Epidemiological findings

The potential index patient was sick from May 21 and died on May 28. Case A took care of index patient during all the 8 days. Case B had visited index case on May 23 and 27. On May 27, both case A and B found bleeding from the mouth, nostrils, and ears of the potential index case. After the death of potential index case, case A and B had cleaned the body and directly touched the blood of the potential index case without any protection.

**Fig. 2:**
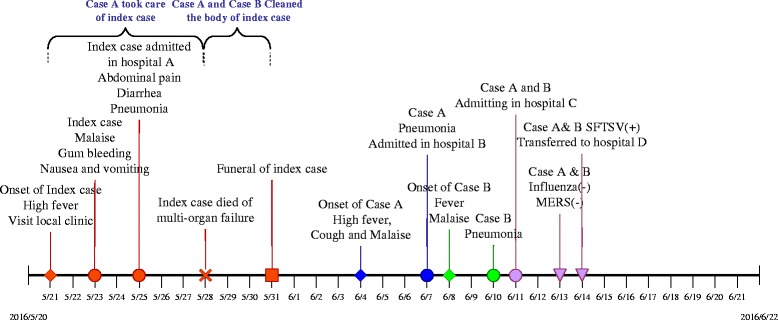
Timeline of key events for the family cluster of SFTS

The potential index patient lived in a village near Tai Lake in Jiangsu Province, which is located to the southeast of China. The village is on a downhill, and she used to climb the hill every day as she had planted some vegetables on the hill. It is unclear whether the patient was bitten by ticks or not. However, investigation on the surroundings of the patient showed that ticks could be found in the village and in other places that the patient came into contact with on the hill. Both case A and case B had their own houses and denied history of tick bite or outdoor activities within 2 weeks before illness onset (Fig. 2).

Altogether, 19 ticks were caught through flagging from the hill and grassland. Eight rodents were caught on the hill, around the village or indoor around the residency of potential index case. Ticks could be found on the surface of rodents. Three out of ten dogs were found to be infected with ticks and tick index was 0.4.

### Close contacts

A total of 32 close contacts including case C were identified in this family cluster. They were mostly relatives from the family. Three close contacts became ill. One was Case C, a 30-year-old female. She was daughter to case B and became ill on June 1, 2016 with the symptoms of coughing and a slight fever. She took some self-prescribed drugs but she could not remember the drug’s name. On June 11, she accompanied her father (case B) and her aunt (case A) to hospital C in Shanghai. She had a fever of 37.8 °C. Blood testing was normal at the time of admission. Rapid testing for influenza A was negative. She was admitted in hospital C and treated with antiviral medicine. She was discharged on June 14 when serum detection for SFTSV was negative. Another relative who became sick was the husband to potential index case’s sister. He got fever on June 8, 2016 and recovered soon without any other symptoms. The third one who was an undertaker and had moved and cleaned the body of the potential index case. He had wiped the blood from the mouth of the potential index case. Other close contacts had no symptoms during the latent period after contact.

### Laboratory testing

On June 13, serum samples were collected from case A and B. On June 14, C_t_ values of case A and B were 32 and 29 respectively in Real-Time PCR assay and both were positive to SFTSV. Sera of close contacts with fever or other symptoms were tested negative by Real Time RT-PCR including case C.

## Discussion

We presented a family cluster of SFTSV imported from Jiangsu province herein. Three family members successively became ill. The potential index case died and two other proven SFTS cases developed a secondary infection following exposure to the potential index case. One secondary case had provided bedside care for potential index case and both the secondary cases had contact with blood from potential index case. As early as 2006, two clusters were suspected to be infected with a novel virus in Anhui province and patients from one cluster were confirmed to be SFTSV positive in 2012 [10]. Another family cluster was retrospectively identified in a hilly area about 110 km south of Nanjing in eastern China in 2007 [8]. In several family cluster reports of SFTSV in China and Korea, evidence of personal contact, especially blood contact was demonstrated [7–12, 18]. Most index patients reported in family cluster were infected through tick bites. Secondary patients in the family possibly became sick by contacting blood infected with SFTS. Genetic susceptibility was supposed to be one of the determinants for susceptibility of family members [19], which might explain why family members were more easily infected. However, person to person transmission was excluded in two family clusters reported in Zhejiang province [20]. In this family cluster, given the facts that two secondary cases had no history of tick bite or outdoor activities on the hill and case A and case B became sick 4 days and 7 days respectively, after the death of potential index case. Person to person transmission was suggested in this family cluster.

Lab-confirmed cases were reported in 16 provinces in China till 2014 [4]. More areas were verified as natural foci of SFTSV. Ticks could serve as a vector and reservoir of SFTSV and mice played a role in SFTSV transmission [21]. Ticks fed on SFTSV-infected mice could acquire the virus and transmit it to other developmental stages of ticks. SFTSV-infected ticks could transmit the virus to mice during feeding. SFTSV genomic RNAs were identified in *Apodemus agrarius* in Zhejiang province [22]. In this family cluster, the residence of index patient was located on a hilly area. Free ticks and mice were found in the surrounding environment and ticks were found feeding on mice. We inferred that SFTSV from ticks on the hill most likely caused the potential index case’s infection though tick bite. However, more evidence will be needed to prove whether SFTSV was endemic in the region near Tai lake or not.

Secondary infection was person-to-person transmission. Two secondary cases had taken care of the potential index case and had close contact with the potential index case. First, two secondary cases had a history of coming into contact with potential index case’s blood and handling or cleaning her corpse, which provided evidence of person-to-person transmission through blood contact. Second, in this family cluster, all three cases developed severe pneumonia at the early stage of infection, which was entirely different from most of other cases of SFTS. We proposed that aerosol person-to-person transmission probably existed in the family cluster. According to previous researches, typical clinical and laboratory manifestations of SFTS included fever, gastrointestinal symptoms, myalgia, chills, thrombocytopenia, and leukopenia [13, 14, 23, 24]. As a first group of serious symptoms, pneumonia was rarely observed in SFTSV infection. Confounded by influenza or avian influenza, it was difficult to be diagnosed. Atypical cases were easily ignored. However, researches had found that exposure to respiratory secretions led to nosocomial transmission of SFTSV among healthcare workers [25]. Positive results were detected in tracheal aspirate and gastric aspirate in an SFTS patient [26]. A cluster report in 2015 showed that SFTSV could be transmitted from person to person, by direct contact and/or aerosol transmission [27]. Our study gave more evidence for aerosol transmission in cluster. In addition, pneumonia may be an early onset symptom which should be explored in future research. One case of secondary-asymptomatic infection was found in a cluster in 2006 in Liaoning province and another asymptomatic case was reported in 2015 in Zhejiang province [28]. More integrated and sensitive clinical characters should be included in SFTS surveillance to identify possible SFTS cases.

## Conclusion

A family cluster of SFTS was confirmed in Shanghai. Two family members were infected by SFTSV most possibly from one clinical SFTS case, who was potential index case for this family cluster. We should establish more surveillance system, within China and in the region, to identify more SFTS cases with atypical symptoms.

## Abbreviations

ALT: Alanine aminotransferase
APTT: Activated partial thromboplastin time
AST: Aspartate aminotransferase
CT: Computerized tomography
pCO_2_: Partial pressure of carbon dioxide
RT-PCR: Real-Time reverse transcription polymerase chain reaction
SFTS: Severe fever with thrombocytopenia syndrome
SFTSV: Severe fever with thrombocytopenia syndrome virus
TB: Tuberculosis
TCO_2_: Total carbon dioxide
